# Extraction of a group-pair relation: problem-solving relation from web-board documents

**DOI:** 10.1186/s40064-016-2864-3

**Published:** 2016-08-05

**Authors:** Chaveevan Pechsiri, Rapepun Piriyakul

**Affiliations:** 1 Department of Information Technology, DhurakijPundit University, Bangkok, Thailand; 2 Department of Computer Science, Ramkhamhaeng University, Bangkok, Thailand

**Keywords:** Word co-occurrence, Elementary discourse unit, Semantic relation, Problem-solving relation

## Abstract

This paper aims to extract a group-pair relation as a Problem-Solving relation, for example a DiseaseSymptom-Treatment relation and a CarProblem-Repair relation, between two event-explanation groups, a problem-concept group as a symptom/CarProblem-concept group and a solving-concept group as a treatment-concept/repair concept group from hospital-web-board and car-repair-guru-web-board documents. The Problem-Solving relation (particularly Symptom-Treatment relation) including the graphical representation benefits non-professional persons by supporting knowledge of primarily solving problems. The research contains three problems: how to identify an EDU (an Elementary Discourse Unit, which is a simple sentence) with the event concept of either a problem or a solution; how to determine a problem-concept EDU boundary and a solving-concept EDU boundary as two event-explanation groups, and how to determine the Problem-Solving relation between these two event-explanation groups. Therefore, we apply word co-occurrence to identify a problem-concept EDU and a solving-concept EDU, and machine-learning techniques to solve a problem-concept EDU boundary and a solving-concept EDU boundary. We propose using k-mean and Naïve Bayes to determine the Problem-Solving relation between the two event-explanation groups involved with clustering features. In contrast to previous works, the proposed approach enables group-pair relation extraction with high accuracy.

## Background

The objective of this research is to extract a semantic relation between two event-explanation groups with concepts and boundary consideration to form a group-pair relation from web-board documents. According to (Khoo and Na 2006), a semantic relation is a directional link between two or more concepts, entities or sets of entities that participate in the relation as follows 〈concept1〉—(*relation*)—〈concept2〉 (where the ‘〈…〉’ and ‘(…)’ symbols represent a concept and a relation type, respectively). The link, which is a dash line, is labeled to indicate the type of relation. For example, the *eat* relation in 〈John〉- - -(*eat*)- - -〈apple〉 can be decomposed into the concept of ‘〈eat〉’ as follows: 〈John〉- - -(*agent*)- - -〈eat〉- - -(*patient*)- - -〈apple〉 where ‘(*agent*)’ and ‘(*patient*)’ are the relation types whilst an ‘agent’ in linguistic typology is an initiator of an event, and a ‘patient’ is an entity undergoing change. Khoo and Na (2006) stated that “concepts and relations are the foundation of knowledge and thought while the concepts are the building blocks of knowledge and the relations are the cement linking up the concepts into the knowledge structures.” (p. 157). The relations and the concepts of knowledge structures are necessary not only for a search engine (Lei et al. 2006), but also for both reasoning and inference in information extraction, information retrieval, question-answering, and text summarization applications through certain web sites (Katrenko et al. 2010).

In much research (Konstantinova 2014; Kim et al. 2009; Girju 2003), the semantic relation determination from texts for various applications mostly relies on the relations, i.e. *is*-*a*, *part*-*of*, and *cause*-*effect*, between two entities of noun phrases without any explanation. Some of the previous researches (Song et al. 2011; Pechsiri and Piriyakul 2010) on knowledge acquisition for reasoning applications attempted to determine the semantic relations, i.e. *disease*-*treatment* and *cause*-*effect*, which are the relations connecting either one entity concept or one event concept without explanation to either a vector of entity concepts or a vector of event concepts as the explanation. However, our research focuses on extracting the group-pair relation as a Problem-Solving relation from web-board documents. The group-pair relation links two event-explanation groups (two vectors of event concepts) where each group is explained by several event concepts, including its boundary determination. Thus, a Problem-Solving relation links a problem-concept group and a solving-concept group. The web-board documents that contain the Problem-Solving relations expressed by experts or practitioners can provide the declarative knowledge and the procedural knowledge for reasoning and inference in other systems of web applications, where the declarative knowledge is “knowing that something is the case/problem” (Hardin 2002, pp. 227) and the procedural knowledge is “knowing how to do something or to solve the problem including motor skills, cognitive skills, and cognitive strategies” (Hardin 2002, pp. 227). Therefore, our research concerns the extraction of the Problem-Solving relation, i.e. a DiseaseSymptom-Treatment relation and a CarProblem-Repair relation, from Thai documents of two domains, a medical-healthcare domain and a car-repair domain, downloaded from **t**he hospital’s web-board on a non-government-organization website (http://haamor.com/) and the car-repair-guru web-boards (https://www.gotoknow.org/posts/113664), respectively for an application with an open source recommendation engine as in the question answering system on the web based system. The Problem-Solving relation on the web-board documents is mostly based on the event explanation with the event semantics of verbs (Pustejovsky 1991) on both the problem-concept group as the problem explanation and the solving-concept group as the solving explanation, described by patients/users and experts, i.e. professional medical practitioners and mechanics. Each medical-healthcare-consulting/car-repair-guru document contains both the disease-symptom-event/carProblem-event explanation and the treatment-event/repair-event explanation, which are expressed in the form of several EDUs [an EDU is an elementary discourse unit, which is a simple sentence/clause defined by Carlson et al. (2003)]. In addition to the solving-event explanation of the Problem-Solving relation, there are two kinds of solution on web-board documents; the actual solution notified by patients/users from their experience, and the recommended solution recorded by experts. For example, each medical-healthcare-consulting document from the web-board contains several EDUs of the symptom concepts along with either the actual-treatment-concept EDUs, followed by the recommended-treatment-concept EDUs or only the recommended-treatment-concept EDUs as shown in the following EDU-Sequence form.

**EDU-Sequence form**
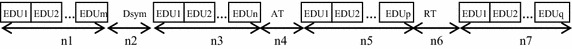
where: Dsym, AT, and RT are a group of disease-symptom-concept EDUs (as a symptom-concept EDU boundary or vector), a group of actual-treatment-concept EDUs (as a treatment-concept EDU boundary or vector), and a group of recommended-treatment-concept EDUs (as a treatment-concept EDU boundary or vector) respectively, as follows:

$$ \begin{aligned} {\text{Dsym}} & = \left( {{\text{EDU}}_{{{\text{sym-}}1}} {\text{EDU}}_{{{\text{sym-}}2}} \ldots {\text{EDU}}_{{{\text{sym-}}a}} } \right)\;{\text{where}}\;{\text{EDU}}_{\text{sym-i}} \;{\text{is}} \; {\text{a}}\;{\text{symptom-concept}}\;{\text{EDU}}, \quad {\text{ i}}  = 1,2, \ldots a, \\ {\text{AT}} & = \left( {{\text{EDU}}_{{{\text{at-}}1}} {\text{EDU}}_{{{\text{at-}}2}} \ldots {\text{EDU}}_{{{\text{at-}}b}} } \right)\;{\text{where}}\;{\text{EDU}}_{\text{at-i}} \;{\text{is}}\;{\text{a}}\;{\text{actual-treatment-concept}}\;{\text{EDU}}, \quad {\text{ i}} = 1,2, \ldots ,b, \\ {\text{RT}} & = \left( {{\text{EDU}}_{{{\text{rt-}}1}} {\text{EDU}}_{{{\text{rt-}}2}} \ldots {\text{EDU}}_{{{\text{rt-}}c}} } \right)\;{\text{where EDU}}_{{{\text{rt-}}i}} \;{\text{is}}\;{\text{a}}\;{\text{recommended-treatment-concept}}\;{\text{EDU}}, \quad {\text{ i}}= 1,2, \ldots ,c \\ \end{aligned} $$

n1–n7 are the number of sequence EDUs and are ≥0 except n2 and n6 which are ≥1.

Therefore, our DiseaseSymptom-Treatment relation can be expressed as follows:

DiseaseSymptom-Treatment Relation: Dsym → AT and Dsym → RT

**Fig. 1:**
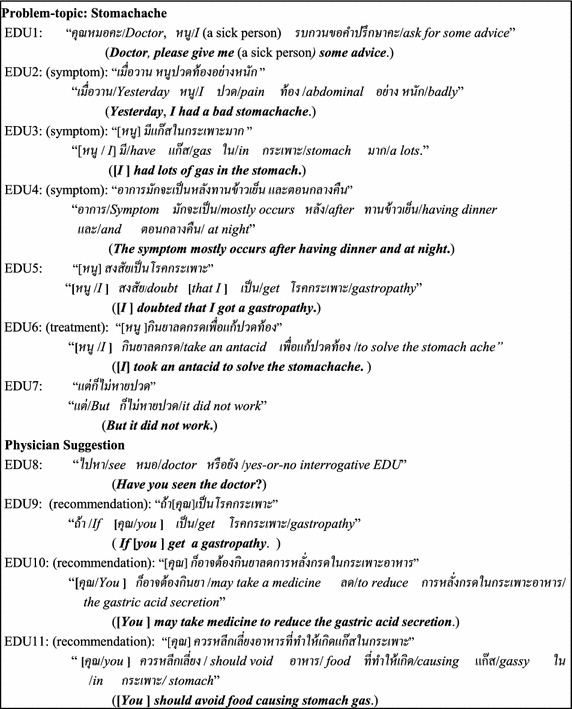
An example of a web-board document showing the DiseaseSymptom–Treatment relation expression (where the […] symbol means *ellipsis*)

The example of the EDU-Sequence form is shown in Fig. 1 where n1 = 1 EDU, n2 = 3 EDUs, n3 = 1EDU, n4 = 1EDU, n5 = 2 EDUs, n6 = 3 EDUs, n7 = 0 EDU, Dsym is EDU2–EDU4, AT is EDU6, and RT is EDU9–EDU11.

**Fig. 2:**
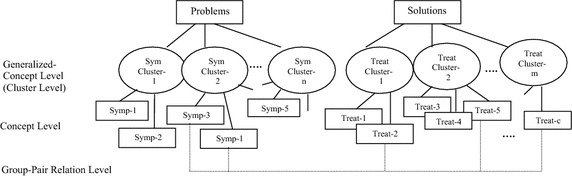
The problem-solving-map representation of the DiseaseSymptom–treatment relation

Moreover, the extracted DiseaseSymptom-Treatment relation from medical-healthcare-consulting documents is represented by a Problem-Solving-Map (PSM), which is the graphical representation of the symptom events with the corresponding treatment events (Fig. 2). The PSM representation helps non-professional people to understand easily how to solve their health problems at the preliminary stage. Thus, the extracted Problem-Solving relation of our research will then benefit the automatic question-answering system on the preliminary problem-solving web-boards while the patients wait for experts.

There are several techniques (Yeleswarapu et al. 2014; Abacha and Zweigenbaum 2011; Fader et al. 2011; Song et al. 2011; Rosario 2005) that have been used to extract the semantic relations of the problems and solutions or effects from documents (see section “Related work”). The group-pair relation as the problem-solving relation in our research is extracted from the downloaded Thai documents of medical-healthcare consultation and carProblem consultation from the hospital web-boards and the car-repair-guru web-boards, respectively. However, the Thai documents have some specific characteristics, such as zero anaphora or implicit noun phrases, without word and sentence delimiters, etc. All of these characteristics are involved in three main problems when extracting the Problem-Solving relation from the web-board documents (see section “Research-problems of problem-solving relation extraction”). The first problem is how to identify a problem-concept EDU, i.e. a symptom-concept EDU and a carProblem-concept EDU, and a solving-concept EDU, i.e. a treatment-concept EDU and repair-concept EDU. The second problem is how to identify the problem-concept EDU boundary, i.e. the symptom-concept EDU boundary (Dsym) and the CarProblem-concept EDU boundary, and the solving-concept EDU boundary, i.e. the treatment-concept EDU boundary (AT/RT) and the repair-concept EDU boundary. In addition, the third problem is how to determine the Problem-Solving relation, i.e. the DiseaseSymptom-Treatment relation and the CarProblem-Repair relation, from the medical-healthcare-consulting documents and the car-repair-guru documents, respectively. To represent these problems, we need to develop a framework which combines a machine learning technique and the linguistic phenomena to learn the several EDU expressions of the Problem-Solving relations. Therefore, we apply a learning relatedness value (Guthrie et al. 1991; Chaudhari et al. 2011) for the words of a word co-occurrence (called “Word-CO”) with a problem concept or a solving concept to identify a problem-concept EDU or a solving-concept EDU. The Word-CO expression in our research is the event expression of two or three adjacent words (after stemming words and eliminating stop words) as a word order pair or a sequence of words existing in one EDU with either a problem concept or a solving concept. The first word of the Word-CO is a verb expression on an EDU with a general Thai linguistic expression (see section “Research-problems of problem-solving relation extraction”) where “verb → verb_strong_ |verb_weak_-noun1| verb_weak_-noun2”. This verb expression can be represented as *v*_*co*_ (*v*_*co*_ → verb_strong_ |verb_weak_-noun1| verb_weak_-noun2). The second word of the Word-CO is the co-occurred word, *w*_*co*_, of *v*_*co*_ and exists immediately after *v*_*co*_, after stemming words and eliminating stop words. Three different machine learning techniques, Maximum Entropy (ME) (Csiszar 1996; Berger et al. 1996; Fleischman et al. 2003), Support Vector Machines (SVM) (Cristianini and Shawe-Taylor 2000), and Logistic Regression Model (LR) (Freedman 2009), are applied to solve the problem-concept EDU boundary and also the solving-concept EDU boundary from the consecutive EDUs. There are two reasons for using these machine learning techniques for the boundary determination; (1) our data on each group of consecutive EDUs (i.e. Dsym as a symptom-concept EDU vector and AT/RT as a treatment-concept EDU vector) are based on a vector of binary features of Word-CO occurrences on the problem-concept EDU vector and the solving-concept EDU vector, and (2) there is a diversity of Word-CO occurrences including some Word-CO occurrences with dependency, where ME is a probabilistic classifier that belongs to the class of exponential models (Csiszar 1996), and SVM is based on the concept of hyperplanes in a multidimensional space that is separated into different class labels (Cristianini and Shawe-Taylor 2000). LR is used to describe data and to explain the relationship between one dependent binary variable and one or more metric (interval or ratio scale) independent variable (Freedman 2009). We also propose using the Naïve Bayes (Mitchell 1997) to determine the Problem-Solving relation from documents after clustering the objects of posted problems on the web-boards and clustering solving features as the feature reduction.

Our research is organized into six sections. In section “Related work”, related work is summarized. Research problems in extracting Problem-Solving relations from documents are described in sections “Research-problems of problem-solving relation extraction”, and A framework for problem-solving relation extraction shows our framework for extracting the Problem-Solving relation. In section “Evaluation and discussion”, we evaluate our proposed model including discussion and then present the conclusion in section “Conclusion”.

## Related work

Several strategies (Yeleswarapu et al. 2014; Abacha and Zweigenbaum 2011; Fader et al. 2011; Song et al. 2011; Rosario 2005) have been proposed to extract a disease treatment relation, a symptom-treatment relation, a drug-adverse-event relation, and other relations from textual data.

Rosario (2005) extracted the semantic relations from bioscience texts. In general, the entities are often realized as noun phrases, and the relationships often correspond to grammatical functional relations, as shown in the following example.

Therefore administration of TJ-135 may be useful in patients with severe acute hepatitis accompanying cholestasis or in those with autoimmune hepatitis.

The disease *hepatitis* and the treatment *TJ*-*135* are entities, and the semantic relation is: *hepatitis*is treated or cured by *TJ*-*135*. The goals of her work are to identify the semantic roles DIS (Disease) and TREAT (Treament), and to identify the semantic relation between DIS and TREAT from bioscience abstracts. She identified the entities (DIS and TREAT) by using MeSH, and the relationships between the entities by using a neural network based on five graphical models with lexical, syntactic, and semantic features. Her results were 79.6 % accurate in the relation classification when the entities were hidden, and 96.9 % when the entities were given.

In 2011 (Abacha and Zweigenbaum 2011) extracted the semantic relations between medical entities (as the treatment relations between a medical treatment and a problem, e.g. a disease symptom) by using a linguistic pattern-based method to extract the relation from selected MEDLINE articles.

$$ {\text{Linguistic}}\;{\text{Pattern}}: \ldots {\text{E}}1 \ldots {\text{be}}\;{\text{effective}}\;{\text{for}}\;{\text{E}}2 \ldots | \ldots {\text{ E1}}\;{\text{was}}\;{\text{found}}\;{\text{to}}\;{\text{reduce}}\;{\text{E}}2 \, \ldots , $$

where E1, E2, or Ei is the medical entity (as well as UMLS concepts and semantic types) identified by MetaMap.

Their treatment relation extraction was based on a couple of medical entities or noun phrases occurring within a single sentence, as shown in the following example:

Fosfomycin (E1) and amoxicillin-clavulanate (E2) appear to be effective for cystitis (E3) caused by susceptible isolates.

Finally, their results showed 75.72 % precision and 60.46 % recall.

Song et al. (2011) extracted the procedural knowledge from MEDLINE abstracts as shown in the following example by using Supporting Vector Machine (SVM) compared to Conditional Random Field (CRF), along with Natural language Processing.

“… 〈*In a total gastrectomy*〉 (Target), 〈*clamps are placed on the end of the esophagus and the end of the small intestine*〉 (P1). 〈*The stomach is removed*〉 (P2) *and* 〈*the esophagus is joined to the intestine*〉 (P3) …”, where P1, P2, and P3 are the solution procedures. They defined procedural knowledge as a combination of the Target and a corresponding solution consisting of one or more related procedures/methods. SVM and CRF were utilized with four feature types: content feature (after word stemming and stop-word elimination) with a unigram and bi-grams in a target sentence, position feature, neighbor feature, and ontological feature to classify the Target. In addition, the other features: word feature, context feature, predicate-argument structure, and ontological feature, were utilized to classify procedures from several sentences. The results were 0.7279 and 0.8369 precisions of CRF and SVM, respectively with 0.7326 and 0.7957 recalls of CRF and SVM, respectively.

Fader et al. (2011) identified the relation between two noun-phrase arguments occurring within one sentence from an open IE (Information Extraction). The open IE contained a massive corpus in which pre-specified vocabulary was not required and the target relations could not be specified in advance. A relation phrase or a verb phrase was then applied to connect the two arguments whilst some relation phrases induced the uninformative and incoherent extractions. To solve this problem, Fader et al. (2011) introduced syntactic constraints and lexical constraints. The syntactic constraints, such as “every multi-word relation phrase must begin with a verb, end with a preposition, and be a contiguous sequence of words in the sentence”, i.e. ‘*has a cameo in*’, ‘*made a deal with*’, etc., can eliminate the problems of uninformative and incoherent extractions. If the relation phrase has too many words, a lexical constraint is used to separate valid relation phrases with a confidence score using a logistic regression classifier. Their precision and recall were 0.8 and 0.62, respectively.

In 2014 (Yeleswarapu et al. 2014) applied the semi-automatic pipeline detection and the extraction of drug-adverse event (drug-AE) pairs from unstructured data, such as user-comment blogs and MEDLINE abstracts, and the structure database (Food and Drug Administration Adverse Event Reporting System). The 12 drugs, diseases and symptoms or adverse events were based on noun phrases, including name entity recognition by using the PubMed dictionary. The Information Component (IC) value by using the Bayesian Confidence Propagation Neural Network is a measure of the disproportionality between entities of the drug-adverse event pairs. The standard deviation for each IC provides a measure of the robustness of the value. The IC is thus a measure of the strength of the dependency between a drug and an AE (Adverse Event). An IC value of zero indicates that there is no quantitative dependency between the drug and AE combinations. If the IC value increases over time and is positive, the positive quantitative association between the drug and the AE is likely to be high. Thus, each extracted drug-AE pair from multiple data sources by Yeleswarapu et al. (2014) implies the relation/association between a certain drug and its adverse events. However, their proposed model extracts the drug-AE pairs from user blogs with less strength of the drug-AE association (based on IC values) than both the MEDLINE abstracts and the adverse event databases.

In most of the previous works, i.e. (Abacha and Zweigenbaum 2011; Rosario 2005), the treatment relation between the medical treatment and the problem (as a disease or a symptom) occurs within one sentence. The drug-AE relation (Yeleswarapu et al. 2014) also occurs within one sentence with several noun phrases including name entities. Furthermore (Fader et al. 2011) worked on the verb phrase as the relation phrase linking two noun-phrase arguments within one sentence whereas Song et al. (2011)’s work could determine several sentences of the treatment method, but there was only one sentence of the problem as the Target disease or symptom. The Problem-Solving relation of this research is a group-pair relation between two groups of several sentences/EDUs, the problem-concept EDU group and the solving-concept EDU group, which result in many Word-CO features with ambiguity, diversity, and dependency occurrences when considering the Problem-Solving relation determination. This research still has another research-problem consideration in which the Problem-Solving relation occurrence and the non-Problem-Solving relation occurrence can occur in the same group pair that has the same problem-concept EDU group and the same solving-concept EDU group. However, the expression of our Problem-Solving relation is based on the event explanation with several EDUs providing more interesting information for people to understand clearly. Therefore, we propose using the Naïve Bayes classifier to determine the Problem-Solving relation from documents where clustering is required to enhance the correct relation extraction. The clustering technique is applied to organize similar problem objects from the problem-concept EDU groups (i.e. symptom-concept EDU vectors and carProblem-concept EDU vectors) and to reduce Word-CO features by clustering the Word-CO features with similar solving concepts to the solving-concept EDU groups (i.e. treatment-concept EDU vectors and repair-concept EDU vectors).

## Research-problems of problem-solving relation extraction

The group-pair relation extraction of this research involves several problems based on the following general Thai linguistic expression of each EDU after stemming words and eliminating stop words:

EDU → : NP1 VP | VP
VP → : verb NP2 | verb adv
verb → : verb_weak_-noun1 | verb_weak_-noun2 | verb_strong_
NP1 → : pronoun | noun1 | noun1 adj | noun1 Phrase
NP2 → : noun2 | noun2 adj | noun2 Phrase | Phrase
Phrase → : AdjPhrase | PrepPhrase
verb_weak_ → : ‘เป็น/be’, ‘มี/have’
verb_strong_ → : ‘รู้สึกปวด/pain’, ‘อาเจียน/vomit’, ‘บวม/swell’,‘ถ่าย/defecate’, ‘รู้สึกแน่น/feel-tight’, ‘กิน/consume’,‘ทา/apply’, ‘ออกกำลัง/exercise’, ‘สั่น/vibrate’, ‘ตาย/fall-down’, ‘มีกำลัง/have power’, ‘หยุด/stop’ ,‘เปลี่ยน/change’, ‘ซ่อม/repair’, ‘ปรับ/adjust’, …
adv → : ‘ยาก/ difficultly’, ‘เหลว/ liquidly’, ‘อย่างแรง/strongly’,…
noun1 → : ‘ ’, ‘แผล/ scar’, ‘ผู้ป่วย/patient’, ‘คน/human’ ,‘อวัยวะ/human-organ’, ‘รถ/car’, ‘ชิ้นส่วนรถ/ car-part’, …
noun2 → : ‘ ’, ‘อาการ/symptom’, ‘ตระคริว/contraction’, ‘สี../..color’, ‘อวัยวะ/human-organ’, ‘ยา/medicine’, ‘อาหาร/food’, ‘กำลัง/power’, ‘เสียง/noise’, …

where NP1 and NP2 are noun phrases, VP is a verb phrase, adv is an adverb, adj is an adjective, AdjPhrase is an adjective phrase, and PrepPhrase is a preposition phrase. For example:

- “*ผู้ป่วยมีอาการแน่นหน้าอก*” (***A patient has a tight chest symptom***.)
  =“(*ผู้ป่วย*/*patient*-noun1)/NP1 (*มี*/*have*-verb_weak_*อาการ*/*symptom*-noun2 *แน่นหน้าอก*/*tight*_*chest*-AdjPhrase)/VP”

(b) “*แผลทีบริเวณนิ้วมือเป็นสีเขียวคล้ำ*” (***A scar at the finger area is dark green color***.)
  = “(*แผล*/*scar*-noun1 *บริเวณนิ้วมือ*/*finger_area* -PrepPhrase)/NP1 (*เป็น*/*is*-verb_weak_*สี*/*color*-noun2 *เขียวคล้ำ*/*dark*-*Green*-adj)/VP”
(c) “*เท้าเป็นแผลผุพอง*” (***The foot has blisters***.)
  = “(*เท้า*/*foot*-noun1)/NP1 (*เป็น*/*is* -verb_weak_*แผล*/*scar*-noun1 *พุพอง*/*blister*-noun2)/VP”
(d) “*คุณยายของหนูรู้สึกปวดหลัง*” (***My grandmother gets back pain***.)
  =“(*คุณยาย*/*grandmother*-noun1 *ของหนู*/*my*-adj)NP1 (*รู้สึกปวด*/*pain*-verb_strong_*หลัง*/*back*- noun2)/VP”
  “[*คุณยาย*] *เวียนศีรษะ*” ([***grandmother***] ***feels dizzy***.)
  = “([*คุณยาย*/*grandmother*]-noun1)/NP1 (*เวียนศีรษะ*/*feel_dizzy*-verb_strong_)/VP”where the […] symbol mean ellipsis.
  “[*คุณยาย*] *ถ่ายเหลว*” ([***grandmother***] ***defecate liquidly***.)
  = “([*คุณยาย*/*grandmother*]-noun1)/NP1 (*ถ่าย*/*defecate*-verb_strong_*เหลว/liquidly*-adv)/VP”
(e) “*ผู้ป่วยกินยาแก้ท้องเสีย”* (***The patient takesan anti*-*diarrhea medicine***.)
  =“(*ผู้ป่วย*/*patient*-noun1)/NP1 (*กิน*/*consume*-verb_strong_*ยา*/*medicine*-noun2 *แก้ท้องเสีย*/*anti*-*diarrhea*- AdjPhrase)/VP”

Therefore, to extract the Problem-Solving relation from documents after passing the pre-processing step of the word-cut and EDU determination, there are three problems that must be solved: how to identify a problem-concept EDU and a solving-concept EDU, how to determine the problem-concept EDU boundary and the solving-concept EDU boundary, and how to determine the Problem-Solving relation from the medical-healthcare-consulting documents and the car-repair-guru documents.

### How to identify problem-concept EDU and solving-concept EDU

According to the corpus behavior study of the medical-healthcare domain and the car-repair domain, most of the symptom/carProblem-concept EDUs and the treatment/repair-concept EDUs are the event expressions expressed by verb phrases. For example:

**Symptom concept**

a) EDU: “*ผู้ป่วยรู้สึกเวียนศีรษะ*” (***A patientfeels dizzy***.)
  “(*ผู้ป่วย*/*A patient*)/NP1 ((*รู้สึกเวียนศีรษะ*/*feel*-*dizzy*)/verb)/VP”
b) EDU: “*ฉันรู้สึกปวดศีรษะ*” (***Ihave a headache***.)
  “(*ฉัน*/*I*)/NP1 ((*รู้สึกปวด*/*pain*)/verb (*ศีรษะ*/*head*)/NP2)/VP”

**CarProblem concept**

c) EDU1: “*เวลา*[*ฉัน*]*แตะเบรก*” (***When*[*I*]*press down on the brake pedal***.)
  “*เวลา*/when ([*ฉัน*/*I*])/NP1 ((*แตะ*/*push*-*down*)/verb (*เบรก*/*brake*-*pedal*)/NP2)/VP”
EDU2: “*เบรกมีเสียงดัง*” (***The brakessqueak***.)
  “(*เบรก*/*brake*)/NP1 ((*มี*/*have*)/verb (*เสียง/noise ดัง*/*loud*)/NP2)/VP”

**Treatment concept**

d) EDU: “*กินยาลดกรด*” (***Take an antacid***.)
  “((*กิน/consume*)/verb (*ยา*/*medicineลดกรด/reduce acid*)/NP2)/VP”

**Repair concept**

e) EDU: “*เช็คผ้าเบรก*” (***Check disc brake pad***.)
  “((*เช็ค/check*)/verb (*ผ้าเบรก/****brake pad***)/NP2)/VP”

However, some verb phrase expressions of the symptom/carProblem concepts are ambiguities. For examples:

f) EDU: “[*คนไข้*]*ถ่ายยาก*” ([***A patient***] ***passed stools with difficulty***.)
  “([*คนไข้*/*patient*])/NP1 ((*ถ่าย*/*defecate*)/verb (*ยาก*/*difficultly*)/adv)/VP”
g) EDU1: “*ห้องน้ำสกปรกมาก*” (***The toilet is very dirty***.)
  “(*ห้องน้ำ*/*toilet*)/NP1 ((*สกปรกมาก*/*is very dirty*)/verb)/VP”
EDU2: “*ฉัน*จึง*ถ่ายยาก*” (***Then, Ipassed stools with difficulty***.)
  “(*ฉัน*/*I*)/NP1 (*จึง*/*then*)/adv ((*ถ่าย*/*defecate*)/verb (*ยาก*/*difficultly*)/adv)/VP”

From (f) and (g) examples, the verb phrase expression of the symptom concept occurs only in (f) with the concept of ‘*ท้องผูก*/*be constipated*’.

h) EDU1: “*รถติดแกสเร่งเครื่องไม่ขึ้น*” (***A car with LPGcan’t accelerate***.)
  “(*รถติดแกส*/*car with LPG*)/NP1 ((*เร่ง*/*accelerate*)/verb (*เครื่อง*/*engine*)/noun2 (*ไม่ขึ้น*/*can’t*)/neg)VP”
i) EDU: “*รถบรรทุกเหล็กเร่งเครื่องไม่ขึ้นบนเนินเขา*” (***A steel truckcan’t accelerate uphill***.)
  “(*รถบรรทุกเหล็ก*/*steel truck*)/NP1 ((*เร่ง*/*accelerate*)/verb (*เครื่อง*/*engine*)/noun2 (*ไม่ขึ้น*/*can’t*)/neg (*บนเนินเขา*/*uphill*)/noun2)/VP”

From the examples h) and i), the verb phrase expression of the carProblem concept occurs only in h) with the concept of ‘*มีปัญหากำลังต่ำ*/*have low power problem*’

This concept-EDU identification problem can be solved by learning the relatedness from two consecutive words on each EDU after stemming words and eliminating stop-words to form the Word-CO of each EDU with the symptom/carProblem concept or the treatment/repair concept. Where the first word of the Word-CO is a verb expression, *v*_*co*_, related to the symptom/carProblem concept or the treatment/repair concept (where *v*_*co*_ ∈ V_*co*_, V_*co*_ = V_*co*1_∪V_*co*2_, V_*co*1_ is a set of verbs related to the symptom/carProblem concepts, and V_*co*2_ is a set of verbs related to the treatment/repair concept set). The second word of the Word-CO is a co-occurred word, *w*_*co*_ (*w*_*co*_ ∈ W_*co*_; W_co_ = W_*co*1_∪W_*co*2_). W_*co*1_ and W_*co*2_ are co-occurred word sets inducing the *v*_*co*1_*w*_*co*1_ co-occurrence and the *v*_*co*2_*w*_*co*2_ co-occurrence to have the symptom/carProblem concept and treatment/repair concept, respectively, where *v*_*co*1_∈ V_*co*1_, *w*_*co*1_∈ W_*co*1_, *v*_*co*2_∈ V_*co*2_ and *w*_*co*2_∈ W_*co*2_. All concepts of V_*co*1_, V_*co*2_, W_*co*1_, and W_co2_ from the annotated corpus are obtained from WordNet (Miller 1995) and MeSH (http://www.ncbi.nlm.nih.gov/mesh).

V_*co*1_ = : {‘*รู้สึกปวด,ปวด*/*pain*’, ‘*คลื่นไส้*/*nauseate*’, ‘*อาเจียน*/*vomit*’, ‘*วิงเวียน*/*be*-*dizzy*’, ‘*หน้ามืด*/*faint*’, ‘*บวม*/*swell*’,‘*ถ่าย*/*defecate*’, ‘*รู้สึกแน่น*/*feel*-*tight*’, ‘*อึดอัด*/*be*-*uncomfortable*’,‘*รู้สึกไม่สบาย*/*be*-*uncomfortable*’, ‘*เบ่ง*/*push*’, ‘*หายใจ*/*breathe*’,*มีอาการ*/*havesymptom*’, ‘*มี*[*อาการ*]/*have*[*symptom*]’, ‘*สั่น*/*vibrate*’, ‘*ตาย*/*fall*-*down*’, ‘*สตาร์ท*/*start*’, ‘*หยุด*/*stop*’,‘*บาง*/*be thin*’,‘*มีกำลัง*/*have power*’, ‘*มีเสียง*/*have noice*’, ‘*ยก*เครื่อง/*overhaul’*, …}
V_*co*2_ = : {‘*กิน*/*consume*’, ‘*ทา*/*apply*’, ‘ใ*ช้*/*apply*’ ‘*รักษา*/*remedy*’, ‘*บำรุง*/*nourish*’, ‘*ลด*/*reduce*’, ‘*ออกกำลัง*/*exercise*’, ‘*ล้าง*/*clean*’, ‘*เช็ค,ตรวจ*/*check*’, ‘*เปลี่ยน*/*change*’, ‘*ซ่อม*/*repair*’, ‘*ปรับ*/*adjust*’, …}
W_*co*1_ = : {‘’, ‘*อวัยวะ*/*human*-*organ*’,‘*ยาก*/*difficultly*’, ‘*ถ่าย*/*stools*’, ‘*เชื้อ*/*germ*’, ‘*เหลว*/*liquidly’*, ‘*อ่อน*/*soft*’, ‘*แรง*/*strong*’, ‘*ประจำเดือน*/*period*’, ‘*แน่นท้อง*/*fullness*’, ‘*ท้องเฟ้อ*/*flatulence*’, ‘*ไข้*/*fever*’, ‘*เครื่องยนต์*/*engine*’, ‘*ต่ำ*/*low*’, ‘*ดัง*/*loud*’, ‘*สำเร็จ*/*successfully*’, …}
W_*co*2_ = : {‘’, ‘*ยา*/*medicine*’, ‘*อาหาร*/*food*’, ‘*อาหารเสริม*/*supplement*’, ‘*รถ*/*car*’, ‘*ชิ้นส่วนรถ*/*car*-*part*’, ‘*เครื่องยนต์*/*engine*’, …}

### How to determine the problem-concept EDU boundary and the solving-concept EDU boundary

According to the medical-healthcare-consulting document shown in Fig. 1, there is no clue (i.e. ‘และ/and’, ‘หรือ/or’, etc.) in both EDU4 and EDU11 to identify the symptom boundary (EDU2–EDU4) and to identify the treatment boundary (EDU9–EDU11), respectively. In addition, in the car-repair-guru documents, there is also no the clue in EDU5 and EDU7 to identify the carProblem-concept EDU boundary (EDU1–EDU5) and the repair-concept EDU boundary (EDU6–EDU7), respectively as shown in the following example.

J) EDU1 (problem): “*เมื่อวาน ผมสตาร์ทรถ*” (***Yesterday he started the car engine***.)
  “*เมื่อวาน*/*Yesterday* (*ผม*/*he*)/NP1 ((*สตาร์ท*/*start*/)*verb (รถ*/*car*)/noun2)/VP”
EDU2 (problem): “*เครื่องมันสั่น*” (***The enginevibrated***.)
  *“*(*เครื่องมัน*/*engine*)NP1 *((สั่น*/*vibrate*)/verb)/VP*”*
EDU3 (problem): “*เหมือนเครื่องจะดับ*” (***It seemed like the engine wouldstop***.)
  “(*เหมือน*/*It seemed like*) (*เครื่อง/engines*)*/*NP1 *((จะดับ/wouldstop*)/verb)*/*VP”
EDU4 (problem): “*เร่งเครื่องไม่ขึ้น*” (***The engine couldn’t beaccelerated***.)
  “((*เร่ง*/*accelerate*)/verb (*เครื่อง*/*engine*)/noun2 (*ไม่ขึ้น*/can’t)/neg)/VP”
EDU5 (problem): “*รถมีกำลังต่ำ*” (***the carhad low power***.)
  “(*รถ*/*car*)/NP1 (*มี*/*have*)verb (*กำลัง/power*)/noun2 (*ต่ำ*/*low*)/VP”
EDU6 (solving): “*เลย*[*ผม*]*เช็คหัวเทียน*” (***Then*[*he*]*checked the spark plug***.)
  “*เลย*/*Then* ([*ผม*/*he*])/NP1 ((*เช็ค*/*check*)/verb (*หัวเทียน*/*spark plug*)/noun2)/VP”
EDU7 (solving): “[*ผม*]*ทำความสะอาดหัวเทียน*” (**[*he*]*cleaned the spark plug***.)
  “([*ผม*/*he*])/NP1 ((*ทำความสะอาด*/*clean*)/verb (*หัวเทียน*/*spark plug*)/noun2)/VP”
EDU8: “[*รถ*] *ก็เป็นปกติ*” (**[*The car*]*then was normal***.)
  “[*The car*]**/**NP1 *ก็*/*then* (*เป็นปกติ/be normal*)/VP”

After the problem-concept EDU and the solving-concept EDU have been identified by using the Word-CO from section “How to identify problem-concept EDU and solving-concept EDU”, we then solve the problem-concept EDU boundary and the solving-concept EDU boundary by applying ME, SVM, and LR to learn a Word-CO pair from the sliding-window size of the two consecutive EDUs with one sliding EDU distance.

### How to determine the problem-solving relation

The relation results of a problem-concept group and a solving-concept group vary between people, i.e. patients, drivers, and other users, even though they have the same problems. For example:

**DiseaseSymptom-treatment relation**

k) EDU1_sym-1_: “*ผู้ป่วยปวดท้องอย่างหนัก*” (***A patienthas a bad stomachache***.)
  “(*ผู้ป่วย*/*patient*)/NP1 ((*ปวด*/*pain*)/verb (*ท้อง*/*abdominal*)/noun2 (*อย่างหนัก*/*badly*)/adv)/VP”
EDU2_sym-2_: “[*เขา*/*He*] *มีแก๊สมากในกระเพาะ*” (**[*He*]*has lots of gas in the stomach.***)
  “[*เขา*/*He*]/NP1 ((*มีแก๊ส/has*gas)/verb (*มาก/a lots*)/adv *ในกระเพาะ*/*insidestomach*)/VP”
EDU3_at-1_: “[*เขา*/*He*] *กินยาลดกรด*” (**[*He*]*takes an antacid***.)
  “[*เขา*/*He*]/NP1 ((*กิน/consume*)/verb (*ยา*/*medicineลดกรด/reduce acid*)/NP2))/VP”
EDU4: “*แต่มันก็ไม่หายปวด*” (***But*[*it*]*does not work***.)
  “แต่/But [มัน/it]/NP1 (ก็ไม่หายปวด/cannot work)/VP”
l) EDU1_sym-1_: “[ผู้ป่วย] ปวดท้อง” (**[A patient] has a stomachache.**)
  “[*ผู้ป่วย*/*patient*]/NP1((*ปวด*/*pain*)/verb (*ท้อง*/*abdominal*)/noun2)/VP”
EDU2_sym-2_: “[*เขา*/*He*] *มีแก๊สในกระเพาะ*” (**[*He*]*has gas in the stomach***.)
  “[*เขา*/*He*]/NP1 ((*มี*/*has แก๊ส*/gas)/verb (*ในกระเพาะ*/*insidestomach*)/PrepPhrase)/VP”
EDU3_at-1_: “[*เขา*/*He*] *กินยาลดกรด*” (**[*He*]*takes an antacid***.)
  “[*เขา*/*He*]/NP1((*กิน/consume*)/verb (*ยา*/*medicineลดกรด/reduce acid*)/NP2))/VP”
EDU4: “[*เขา*/*He*] *รู้สึกดีขึ้น*” ([***He*]*feels better***.)
  “[*เขา*/*He*]/NP1 ((*รู้สึกดีขึ้น*/*feel better*)/verb)/VP”

According to the examples k) and l), the DiseaseSymptom-Treatment relation occurs only on l) because EDU4 of l) contains ‘*รู้สึกดีขึ้น*/*feel better*’ as Class-cue-word (see section “Corpus preparation”) of the Problem-Solving relation.

**CarProblem-Repair relation**

m) EDU1(problem): “*รถของผมสตาร์ทไม่ติด*” (***My car can’t bestarted successfully***.)
  “(*รถของผม*/*my car*)/NP1 (*ไม่สามารถ*/*can’t สตาร์ท*/*bestartedติด/successfully*)/VP”
EDU2 (problem): “*เมื่อเครื่องร้อนเท่านั้น*” (***when the engineis hot***.)
  “*เมื่อ*/*when* (*เครื่อง*/*engine*)/NP ((*ร้อน*/*be hot*)/verb *เท่านั้น*/*only*)/VP”
EDU3 (solving): “*ผมเพิ่งเปลี่ยนแบต*” (***I have justchange the battery***.)
  “(*ผม*/*I*)/NP1 (*เพิ่ง*/*just* (*เปลี่ยน*/*change*)/verb (*แบต*/*battery*)/noun2)/VP”
EDU4 (solving): “*ล้างหัวเทียน*” (***Clean the spark plug***.)
  “((*ล้าง*/*clean*)/verb (*หัวเทียน*/*spark plug*)/noun)/VP”
EDU5 (solving): “*ผมเลยเปลี่ยนไดสตาร์ท*” (***I thenchanged the starter***.)
  “(*ผม*/*I*)/NP1 *เลย*/*then* (*เปลี่ยน*/*change*)/verb (*ไดสตาร์ท*/*starter*)/noun2)/VP”
EDU6: “*มันก็ไม่สามารถแก้ได้*” (***It then can’t be fixed***.)
  “(*มัน*/*it*)/NP1 (*ก็*/*then ไม่สามารถ*/*can’t แก้ได้*/*be fixed*)/VP”
n) EDU1 (problem): “*เครื่องร้อนสตาร์ทไม่ติด*” (***The hot engine can’t be started successfully***.)
  “(*เครื่องร้อน*/*hot engine*)/NP1 (*ไม่สามารถ*/*can’t* (*สตาร์ท*/*start*)/verb (*ติด*/*successfully*)/Adv)/VP”
EDU2 (problem): “*รอสักพัก*” (***Waitfor a while***.)
  “((*รอ*/*wait*)/verb *สักพัก*/*for awhile*)/VP”
EDU3 (problem): “[*มัน*]*จึงสามารถสตาร์ทติด*” (**[*It*]*then canbe started successfully***.)
  [*มัน*/*It*]/NP1 *จึง*/*then* ((*สามารถ*/*can* (*สตาร์ท*/*start*)/verb (*ติด*/*successfully*)/Adv)/VP
EDU4 (solving): “*แบตและหัวเทียนปกติ*” (***the battery andthespark plug are normal***.)
  “(*แบต*/*battery และ*/*and หัวเทียน*/*spark plug*)/NP1 (*ปกติ*/*be normal*)/verb)/VP”
EDU5 (solving): “*เลยเปลี่ยนไดสตาร์ท*” (***Thenchange the starter***.)
  “(*เลย*/*then* (*เปลี่ยน*/*change*)/verb (*ไดสตาร์ท*/*starter*)/noun2)/VP”
EDU6: “*ตอนนี้การสตาร์ทรถเป็นปกติ*” (***Now starting car isnormal***.)
  “*ตอนนี้*/*Now* (*การสตาร์ท*/*starting รถ*/*car*)/NP1 (*เป็นปกติ*/*b*e *normal*)/VP”

According to examples m) and n), the CarProblem-Repair relation occurs only on n) because EDU6 of n) contains ‘*เป็นปกติ*/*b*e *normal*’ as Class-cue-word of the Problem-Solving relation.

Therefore, we propose automatically learning the Problem-Solving relation in documents by using the Naïve Bayes classifier, with clustering objects from several symptom/carProblem-concept EDU vectors and clustering features as the feature reduction of all features from treatment/repair-concept EDU vectors. Where each symptom/carProblem-concept EDU and each treatment/repair-concept EDU are represented by the Word-CO with the symptom/carProblem concept, *v*_co1_*w*_co1_, the Word-CO with the treatment/repair concept, *v*_co2_*w*_co2_, respectively. Each symptom/carProblem-concept EDU boundary and each treatment/repair-concept EDU boundary is represented by a symptom/carProblem-concept EDU vector, 〈*v*_co1-1_*w*_co1-1_, *v*_co1-2_*w*_co1-2_, …, *v*_co1-*a*_*w*_co1-*a*_〉, and a treatment/repair-concept EDU vector, 〈*v*_co2-1_*w*_co2-1_, *v*_co2-2_*w*_co2-2_, …, *v*_co2-*b/c*_*w*_co2-*b/c*_〉, respectively.

## A framework for problem-solving relation extraction

**Fig. 3:**
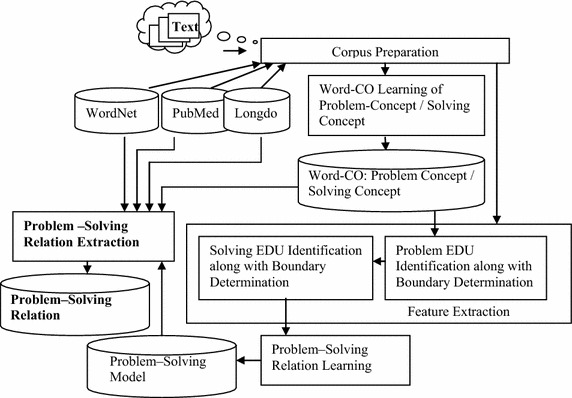
System overview where the input is text or downloaded documents and the output is the problem-solving relation i.e. a DiseaseSymptom–treatment relation and a CarProblem–repair relation

There are five steps in our framework. The first step is the corpus preparation step followed by the step of Word-CO concept learning, especially problem concepts (i.e. symptom/carProblem concepts) and solving concepts (i.e. treatment/repair concepts). The feature extraction step for the Problem-Solving relation learning step is then carried out, which is followed by the Problem-Solving relation extraction step as shown in Fig. 3.

### Corpus preparation

**Fig. 4:**
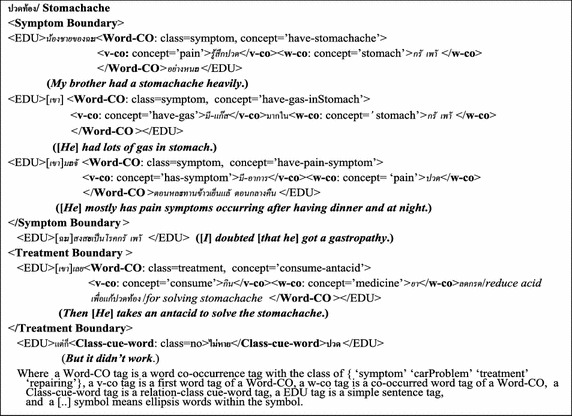
DiseaseSymptom–treatment relation annotation

This step is the preparation of a medical-healthcare corpus and a car-repair corpus in the form of EDUs from the medical-healthcare-consulting documents and the car-repair documents downloaded from the hospital web-board (http://haamor.com/) and the car-repair-guru web-board (https://www.gotoknow.org/posts/113664, http://pantip.com/topic/31660469), respectively. The step involves using Thai word segmentation tools (Sudprasert and Kawtrakul 2003), including named entities (Chanlekha and Kawtrakul 2004). After the word segmentation is achieved, EDU segmentation is then dealt with (Chareonsuk et al. 2005). Thus, there are 6000 EDUs in the medical-healthcare corpus and 2000 EDUs in the car-repair corpus. The medical-healthcare corpus consists of three disease categories with 2000 EDUs in each disease category, i.e. a Gastro-intestinal disease, a Heart-Brain disease, and a Childhood disease. These corpora are separated into 2 parts; a learning part (4500 EDUs from the medical-healthcare-consultation documents and 1500 EDUs from the car-repair documents) and an evaluation part (1500 EDUs from the medical-healthcare-consultation documents and 500 EDUs from the car-repair documents). The learning part is used to learn the Word-CO concepts, the boundaries (the problem-concept EDU boundary and the solving-concept EDU boundary), and the Problem-Solving relation, based on tenfold cross validation. The evaluation part is used to test or evaluate the feature extraction (as the correct boundary determination) and the Problem-Solving relation extraction (see section “Evaluation and discussin”). In addition to this step, the corpus semi-automatically annotates the Word-CO concepts of the problem concepts and the solving concepts along with Class-cue-word annotation to specify the cue word of the Problem-Solving relation with the Class-type set {“yes”, “no”} as shown in Fig. 4 as an example of the Problem-Solving relation annotation. All the concepts of the Word-CO refer to WordNet (http://word-net.princeton.edu/obtain) and MeSH after translating from Thai to English, by using Lexitron (the Thai-English dictionary) (http://lexitron.nectec.or.th/).

### Word-CO concept learning

According to Guthrie et al. (1991), Chaudhari et al. (2011), the relatedness value, *r*, was applied in this research to indicate the relatedness between two consecutive words of the Word-CO, *v*_*coi*_*w*_*coi*_ from the annotated corpora after stemming words and eliminating stop words, with either the problem concept (i.e. a symptom/carProblem concept) or the solving concept (i.e. a treatment/repair concept) as shown in Eq. (1). Where each *v*_*coi*_*w*_*coi*_ existing on several EDUs of documents has a relatedness r(*v*_*coi*_, *w*_*coi*_) value with either a positive or a negative concept. For example, if *v*_*coi*_ is *v*_*co*1_, one relatedness value of a *v*_*co*1_*w*_*co*1_ occurrence is the problem concept (i.e. a symptom/carProblem concept) as the positive concept. Another relatedness value of the same *v*_*co*1_*w*_*co*1_ occurrence is the non-problem concept (i.e. a non-symptom/non-carProblem concept) as the negative concept. If *v*_*coi*_ is *v*_*co*2_, one relatedness value of a *v*_*co*2_*w*_*co*2_ occurrence is the solving concept (i.e. a treatment/repair concept) as the positive concept. Another relatedness value of the same *v*_*co*2_*w*_*co*2_ occurrence is the non-solving concept (i.e. a non-treatment/non-repair concept) as the negative concept. Only the *v*_*coi*_*w*_*coi*_ occurrence of the positive concept (the problem concept or the solving concept) with a higher r(*v*_*coi*_, *w*_*coi*_) value than the one of the negative concept (the non-problem concept or the non-solving concept) is collected as an element of VW_problem_ or VW_solving_ respectively. Where *v*_*co*1_*w*_*co*1_∈VW_problem_; VW_problem_ is a set of Word-COs with the problem concepts, and *v*_*co*2_*w*_*co*2_∈ VW_solving_;VW_solving_ is a set of Word-COs with the solving concepts. VW_problem_ and VW_solving_ are used to identify the problem concept EDU and the solving concept EDU, respectively.

1 $$ r\left( {v_{coi} ,w_{coi} } \right) = \frac{{fv_{coi} w_{coi} }}{{fv_{coi} + fw_{coi} - fv_{coi} w_{coi} }}. $$

where $r\left( {v_{coi} ,w_{coi} } \right)$ is the relatedness of Word-Co with a problem/symptom concept if *coi* = *co*1 or a solving/treatment concept if *coi* = *co*2.

$v_{coi} \in V_{coi} ,w_{coi} \in W_{coi}$*V*_*co*1_ is a set of verbs with the problem/symptom concepts. *V*_*Co*2_ is a set of verbs with the solving/treatment concepts. *W*_*co*1_ is the co-occurred word set having the problem/symptom concept in the *v*_*co*1_*w*_*co*1_ co-occurrence. *W*_*co*2_ is the co-occurred word set having the solving/treatment concept in the *v*_*co*2_*w*_*co*2_ co-occurrence. *fv*_*coi*_ is the number of *v*_*coi*_ occurences. *fw*_*coi*_ is the number of *w*_*coi*_ occurences. *fv*_*coi*_*w*_*coi*_ is the number of *v*_*coi*_ and *w*_*coi*_ occurences.

### Feature extraction

This step involves the extraction of two feature groups, a problem feature group and a solving feature group, to learn the Problem-Solving relation in the next step, for example, the feature extraction on the medical-healthcare domain; the problem feature group is the symptom-concept EDU boundary (Dsym represented by a symptom-concept EDU vector, 〈*v*_co1-1_*w*_co1-1_, *v*_co1-2_*w*_co1-2_, …, *v*_co1-*a*_*w*_co1-*a*_〉) and the solving feature group is the treatment-concept EDU boundary (AT/RT represented by a treatment-concept EDU vector, 〈*v*_co2-1_*w*_co2-1_, *v*_co2-2_*w*_co2-2_, …, *v*_co2-*b/c*_*w*_co2-*b/c*_〉). Therefore, after the starting EDU of the problem-concept EDU boundary and the solving-concept EDU boundary have been identified by *v*_*coi*_*w*_co*i*_ from VW_problem_ and VW_solving_, the problem-concept EDU boundary (i.e. Dsym) and the solving-concept EDU boundary (i.e. AT/RT) are determined by each of the following techniques: ME, SVM, and LR, along with sliding the window size of two adjacent EDUs with one EDU distance. (Where *coi* = *co*1, *v*_*co*1_*w*_*co*1_ is Word-CO with a symptom/carProblem concept called a “symptom/carProblem Word-CO” or “Problem Word-CO”, and *coi* = *co*2, *v*_*co*2_*w*_*co*2_ is Word-CO with a treatment/repair concept called a “treatment/repair Word-CO” or “Solving Word-CO”)

ME (Csiszar 1996; Berger et al. 1996; Fleischman et al. 2003) can be used as the classifier of the r class when the probability p(*r|x*) is the argmax p(*r|x*) to determine either the Dsym boundary classes or the AT/RT boundary classes as shown in Eq. (2). Where *r* is the Dsym boundary classes or the AT/RT boundary classes (the boundary is ending if *r* = 0, otherwise *r* = 1), and *x* is the binary vector of Word-CO (*v*_*coi*_*w*_*coi*_) features containing all Word-CO pairs, *v*_*coi*-*j*_*w*_*coi*-*j*_*v*_*coi*-*j*+1_*w*_*coi*-*j*+1_. According to Eq. (2), both λ_*l*_ of each *v*_*co*1-*j*_*w*_*co*1-*j*_ and λ_*l*_ of each *v*_*co*2-*j*_*w*_*co*2-*j*_ are the results from the supervised learning of ME by sliding the window size of two adjacent EDUs with one EDU distance through the problem/symptom-concept EDU boundary and through the solving/treatment-concept EDU boundary, respectively. Then, all λ_*l*_ of *v*_*co*1_*w*_*co*1_ and all λ_*l*_ of *v*_*co*2_*w*_*co*2_ from the ME learning are used to determine and extract Dsym and the AT/RT, respectively from the testing corpus with Eq. (2).

2 $$ \begin{aligned} p(r|x) & = \mathop {\arg \hbox{max} }\limits_{r} \frac{1}{z}\exp \left( {\sum\limits_{l = 1}^{n} {\lambda_{l} f_{yes,coi\_j,l} \left( {r,v_{coi\_j} w_{coi\_j} } \right)} + \sum\limits_{l = 1}^{n} {\lambda_{l} f_{no,coi\_j,l} \left( {r,v_{coi\_j} w_{coi\_j} } \right)} } \right. \\ \left. {\quad + \sum\limits_{l = 1}^{n} {\lambda_{l} f_{yes,coi\_j + 1,l} \left( {r,v_{coi\_j + 1} w_{coi\_j + 1} } \right)} + \sum\limits_{l = 1}^{n} {\lambda_{l} f_{no,coi\_j + 1,l} \left( {r,v_{coi\_j + 1} w_{coi\_j + 1} } \right)} } \right) \\ \end{aligned} $$

where $v_{coi\_j + 1} w_{coi\_j} \in {\text{VW}}_{problem} \;and\;v_{coi\_j + 1} w_{coi\_j + 1} \in {\text{VW}}_{problem}$*ifcoi* = *co*1 *and* VW_*probelm*_*is a set of Work*-*CO with the problem/symptom concepts*. $v_{coi\_j + 1} w_{coi\_j} \in {\text{VW}}_{solving} \;and\;v_{coi\_j + 1} w_{coi\_j + 1} \in {\text{VW}}_{solving}$*ifcoi* = *co*2 *and* VW_*solving*_*is a set of Work*-*CO with the solving/treatment concepts*.

SVM (Cristianini and Shawe-Taylor 2000) with the linear kernel: The linear function, f(x), of the input x = (x_1_…x_n_) assigned to the positive class if f(x) ≥0, and otherwise to the negative class if f(x) < 0, can be written as

3 $$ \begin{aligned} f({\text{x}}) = \langle {\text{w}} \cdot {\text{x}}\rangle + b \hfill \\ = \sum\limits_{j = 1}^{n} {w_{j} } x_{j} + b \hfill \\ \end{aligned} $$

where x is a dichotomous vector number, w is a weight vector, *b* is a bias, and (w,*b*)∈ R^*n*^ × R are the parameters that control the function. The SVM learning is to determine *w*_*j*_ and b for each Word-CO feature, *v*_*coi*-*j*_*w*_*coi*-*j*_ (*x*_*j*_) in each Word-CO pair, *v*_*coi*-*j*_*w*_*coi*-*j*_*v*_*coi*-*j*+1_*w*_*coi*-*j*+1_, from the supervised learning of SVM by sliding the window size of two consecutive EDUs with one sliding EDU distance where *j* = 1, 2, …, *n* and *n* is End-of-Boundary. The weight vector of all *v*_*co*1-*j*_*w*_*co*1-*j*_ and the weight vector of all *v*_*co*2-*j*_*w*_*co*2-*j*_ from the SVM learning were used to determine the boundary of Dsym and the boundary of AT/RT, respectively from the testing corpus with Eq. (3)., All Dsym features and all AT/RT features are then extracted for the Problem-Solving/DiseaseSymptom-Treatment relation learning.

LR (Freedman 2009): The logistic regression model of the research is based on the linear logistic regression with binary vector data. The distinguishing feature of the logistic regression model is that the variable is binary or dichotomous. Usually, the input data with any value from negative to positive infinity would be used to establish which attributions are influential in predicting the given outcome with values between 0 and 1, and hence is interpretable as a probability. The logistic function can be written as:

4 $$ F(x) = \frac{1}{{1 + e^{{ - (\beta_{0} + \beta_{1} x_{1} + \beta_{2} x_{2} )}} }} $$

*F*(*x*) is interpreted as the probability of the given outcome to be predicted where *x*_1_ and *x*_2_ are attribute variables and *ß*_0_, *ß*_1_, and *ß*_2_ are the model estimators which play the role of momentum for each attribute. The research applies Eq. (4) to extract the features within each boundary (Dsym, AT/RT) with *F*(*x*) interpreted as the probability of either “Continue” as the “C” class or “End-of-Boundary” as the “E” class by the following rules.

Rule1(C-Class): If (*F*(*x*)_C-Class_ >= 0.5 then “Continue” (sliding two consecutive EDUs)

Rule2(E-Class): If (*F*(*x*)_E-Class_ >= 0.5 then “End-of-Boundary”(stop sliding two EDUs)

5 $$ Boundary - Deter\hbox{min} ation \, = \, Max\left( {F\left( x \right)_{C - Class} ,F\left( x \right)_{E - Class} } \right) $$

where *x*_1_ and *x*_2_ are the attribute variable pair of each Word-CO pair, *v*_*coi*-*j*_*w*_*coi*-*j*_*v*_*coi*-*j*+1_*w*_*coi*-*j*+1_, of each EDU pair from the supervised learning of LR in Eq. (5) by sliding the window size of two adjacent EDUs with one sliding EDU distance where *j* = 1,2,..,*n* and *n* is End-of-Boundary.

### Problem-solving relation learning

The Problem-solving relation occurrence on documents in this research contains several problem EDUs and several solving EDUs, which result in several problem-Word-CO features and several solving-Word-CO features, i.e. 197 different symptom-Word-CO features and 118 different treatment-Word-CO features. Hence, the research enhances the correct Problem-Solving relation determination by applying a clustering technique to group the similar problem objects and to reduce the solving-concept Word-CO features as the feature reduction before learning the Problem-Solving relation. The research clustered the n samples of the posted problems on the web-board by using k-mean as shown in Eq. (6) (Aloise et al. 2009) where k_1_ is the number of k-clusters for the problem object clustering and k_2_ is the number of k-clusters for the solving feature clustering. k_1_ and k_2_ are predefined from 2 to 10. The expert then select k_1_ = 6, k_2_ = 7 and k_1_ = 5, k_2_ = 6 for the DiseaseSymptom-Treatment relation learning and the CarProblem-Repair relation learning, respectively.

6 $$ Cluster({\text{x}}_{j} ) = \mathop {\mathop {\arg \hbox{min} }\limits_{1 \le k \le K} }\limits_{{}} \left\| {{\text{x}}_{j} } \right. - \left. {\mu_{k} } \right\|^{2} $$

where x_*j*_ is a problem-concept EDU vector, i.e. Dsym, of an object 〈*v*_*co*1-1_*w*_*co*1-1_, *v*_*co*1-2_*w*_*co*1-2_, …, *v*_*co*1-*a*_*w*_*co*1-*a*_〉 and *j* = 1, 2, …, *n* posted problems. *μ*_k_ is the mean vector of the kth cluster. The highest number of *v*_*co*1-*i*_*w*_*co*1-*i*_ occurrences in each cluster is selected as its cluster representative. For example, the symptom cluster set (Y) {rhinorrhoea-based-cluster, abdominalPain-based-cluster, brainSymptom-based-cluster, …, nSymptom-based-cluster} is obtained in this research.

Equation (6) is replaced x_*j*_ with *x*_*j*_ to cluster the solving features, i.e. AT/RT, where *x*_*j*_ is a Word-CO element. For example, *x*_*j*_ is a Word-CO element, *v*_*co*2-*i*_*w*_*co*2-*i*_, of AT ∪ RT and *j* = 1, 2, …, *m*Word-COs, *v*_*co*2_*w*_*co*2_. After clustering the treatment features, the highest number of the general concept (based on WordNet and MesH) of *v*_*co*2-*i*_*w*_*co*2-*i*_ occurrences in each cluster is selected as its cluster representative. The treatment cluster set (Z) {relax-based-cluster, foodControl-based-cluster, injectionControl-based-cluster, …, mTreatment-based-cluster}is then obtained in this research.

With regard to clustering the extracted feature vectors from section “Feature extraction”, the Problem-Solving relation, i.e. the DiseaseSymptom-Treatment relation, is learnt by using Weka to determine the probabilities of *y*_*1*_, …, *y*_*a*_, *z*_*1*_, …, *z*_*h*_ with the Class-type set of the DiseaseSymptom-Treatment relation,{‘yes’ ‘no’} where *y*_*1*_, …, *y*_*a*_ ∈Y, *z*_*1*_, …, *z*_*h*_∈Z, and *h* is max(*b*, *c*) from AT and RT. The Class-type set is specified on any five EDUs right after AT or RT. An element of the Class-type set is determined from the following set of Class-cue-word patterns.

Class-cue-word pattern = {‘**cue**:*หาย*/*disappear* = **class**:yes’, ‘**cue**:*รู้สึกดีขึ้น*/*feel better* = **class** :yes’, ‘cue:ไ*ม่ปวด*/*do not pain* = **class**:yes’, ‘**cue:**“” = **class**:yes’, ‘**cue**:*ไม่หาย*/*appea*r = **class**: no’, ‘**cue**:*ยังปวดอยู่*/*still* pain = **class**:no’, ‘**cue**:*ปวดมากขึ้น*/*have more pain* = **class**: no’, …}

### Problem-solving relation extraction

**Fig. 5:**
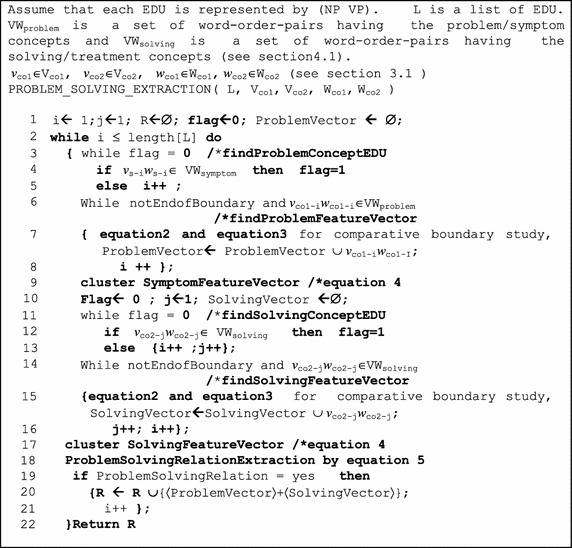
The problem-solving relation extraction algorithm to extract the DiseaseSymptom–treatment relation

The objective of this step is to recognize and extract the Problem-Solving relation from the test corpus by using Naïve Bayes. For example, the DiseaseSymptom-Treatment relation extraction by Naïve Bayes is shown in Eq. (7) with probabilities of *y*_*1*_, …, *y*_*a*_, *z*_*1*_*, …, z*_*h*_ from the previous step with the algorithm shown in Fig. 5.

**Fig. 6:**
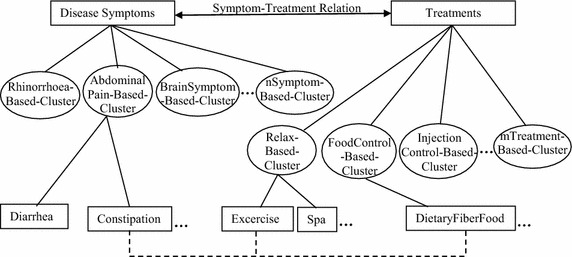
The PSM representation of the DiseaseSymptom–treatment

Moreover, the extracted DiseaseSymptom-Treatment relation of this step can be used to construct PSM as shown in Fig. 6.

7 $$ \begin{aligned} SymTreat\_\text{Re} lClass & = \mathop {\arg \hbox{max} }\limits_{class \in Class} P(class|y_{1} ,y_{2} , \ldots ,y_{a} ,z_{1} ,z_{2} ,..,z_{h} ,dt) \\ = \mathop {\arg \hbox{max} }\limits_{class \in Class} P(y_{1} |class)P(y_{2} |class) \ldots P(y_{a} |class)P(z_{1} |class) \\ \quad P(z_{2} |class) \ldots P(z_{h} |class)P(dt|class)P(class) \\ \end{aligned} $$

where *y*_1_, *y*_2_, … *y*_*a*_ ∈ *Y*, *Y is a problem/symptom cluster set. z*_1_, *z*_2_, … *z*_*h*_ ∈ *Z*, *Z is a solving/treatment cluster set. dt* = *DiseaseTopic Class* = *{“yes”, “no”}*

## Evaluation and discussion

The test corpora of 2000 EDUs employed to evaluate the proposed methodology for extracting the group-pair relation between two event-explanation groups as in the Problem-Solving relation, i.e. the DiseaseSymptom-Treatment relation and the CarProblem-Repair relation, is collected from the downloaded medical-healthcare-consulting documents and the downloaded car-repair-guru documents from the hospital’s web-boards and the car-repair-guru web-boards, respectively. The test corpora, which consist of 500 EDUs for each disease category (Gastro-intestinal disease, Heart-Brain disease, and Childhood disease) and 500 EDUs for the GeneralCar-Problem category are used to test or evaluate the feature extraction and the Problem-Solving relation extraction based on three experts with max win voting. Each category of the test corpora holds on average of 30 posted problem-solving documents with several topic names. The feature extraction as Problem Word-CO occurrences and Solving Word-CO occurrences is evaluated as the problem EDU identification and solving EDU identification, respectively. The feature extraction is also evaluated as the boundary determination of a problem-concept EDU boundary, i.e. Dsym and a carProblem-concept EDU boundary, and a solving-concept EDU boundary, i.e. AT/RT and a repair-concept EDU boundary. The evaluations of the Problem Word-CO identification and the Solving Word-CO identification are based on the precision and the recall of using VW_problem_ and VW_solving_ to identify the problem-concept EDUs and the solving-concept EDUs, respectively. In addition, the results of using three different models (ME, SVM, and LR) for the learning boundary of each EDU group (a problem-concept EDU group and a solving-concept EDU group) are evaluated by the correctness percentage of the EDU boundary determination (see Table 1).

**Table 1:** The accuracy of word-co identification and the accuracy of boundary determination

Disease categoriesand car-problem category (500 EDUs per category)	# of different problem word-CO	# of different solving word-CO	Correctness of problem word-CO Identification		Correctness of solving word-CO Identification		% Correctness of determining boundary of each EDU group; i.e. Dsym, AT/RT					
			Precision	Recall	Precision	Recall	Problem-Concept-EDU boundary			Solving-Concept-EDU boundary		
							ME	LR	SVM	ME	LR	SVM
Childhood-disease	74	39	0.893	0.762	0.882	0.857	80.8	82.1	81.5	91.7	90.4	89.7
Abdominal disease	73	41	0.875	0.700	0.913	0.848	80.0	81.8	80.9	87.8	87.5	87.1
Heart/brain disease	50	38	0.901	0.846	0.894	0.850	81.6	85.5	85.0	89.4	89.0	88.6
GeneralCar-problem	37	68	0.881	0.804	0.906	0.894	*92.3*	91.9	91.3	87.5	88.7	88.3

Italic value indicates the highest achieved %correctness among all experiments

From Table 1, the average precision of using VW_problem_ and VW_solving_ to identify the symptom concept EDUs and the treatment concept EDUs are 0.889 and 0.896, respectively, with average recalls of 0.769 and 0.852, respectively. The reason for having low recall of the symptom-concept-EDU identification is that a Word-CO with two adjacent words after the stop-word removal and stemming words as *v*_*co*1_*w*_*co*1_ is insufficient to cover the symptom concept, i.e. ‘*รู้สึก*/*feel มี/there is อะไร*/*something กดทับ*/*pressing on หน้าอก*/*chest*’ (***feel tight chest***). Moreover, there is Cause-Effect relation occurrence which involves the problem Word-CO occurrence and results in reducing the precision of the symptom-concept-EDU identification by incorrectly identifying the symptom-concept EDUs as shown in the following topic name of the AbdominalDisease category (where the problem Word-CO occurrence, ‘***has*** + ***diarrhea***’, of EDU_at-1_ is an effect from taking the flatulence relief medicine but is not an abdominal disease symptom).

Topic name: “*ปวดท้องและไม่สบายท้อง*/***Have a Stomachache & Abdominal Discomfort***”
EDU_sym-1_: “[*คนไข้*/*A patient*]*/*NP1 ((*มี*/*has*)/verb (*อาการปวดท้อง*/*stomachache*)/NP2 *มาหลายวัน*/*for several days*)/VP” the Problem Word-CO
  ([***A patient***] ***hada stomachachefor several days***.)
EDU_sym-2_: “[*คนไข้*/*A patient*]/NP1 ((รู้*สึก*/*feel***)/**verb (*แน่นท้อง*/*fullness*)/NP2)/VP”
  ([***A patient***] ***feelsfullness***.)
EDU_at-1_as Effect: “แล้ว/*Then* [*คนไข้*/*A patient*]/NP1 ((*มี*/*has*)/verb *อาการท้องเสีย*/*diarrhea*)/NP2)/VP”
  (***Then*** [***A patient***] ***hasdiarrhea***.)
EDU_at-2_as Cause: “(เนื่องจาก/*because*)/Conj [*คนไข้*/*A patient*]/NP1 ((*กิน*/*takes*)/verb (*ยาแก้ท้องอืด*/*flatulence relief medicine*)/NP2)/VP”
  (***because*** [***A patient***] ***takesflatulence relief medicine***.)

Table 1 also shows two boundary evaluations of the problem-concept group (the problem-concept EDU boundary) and the solving-concept group (the solving-concept EDU boundary) in two different domains, a medical-healthcare domain and a car-repair domain. According to the disease categories in Table 1, each disease category shows that the number of different Problem Word-CO occurrences (the diversity of Problem Word-CO occurrence) is higher than the number of different Solving Word-CO occurrences (the diversity of Solving Word-CO occurrence). The Word-CO occurrence diversity resulting in the Word-CO occurrence frequency allows this research to learn the boundary of each event-explanation group, i.e. Dsym and AT/RT, by ME, SVM, and LR after using VW_problem_ and VW_solving_ to identify the problem-concept EDUs and the solving-concept EDUs, respectively. From Table 1, the frequency of Word-CO occurrences affects the %correctness of the boundary determination by ME. For example, the result of the boundary determination by ME for the disease categories is that the %correctness of solving-concept-EDU-boundary determination is higher than the %correctness of the problem-concept-EDU-boundary determination where each disease category has low diversity (high frequency) of the solving/treatment Word-CO occurrence and high diversity (low frequency) of the problem/symptom Word-CO occurrence. In addition, the result of the boundary determination by ME for the car-problem category is that the %correctness of the problem-concept-EDU-boundary determination is higher than the %correctness of the solving-concept-EDU-boundary determination where the car-problem category has low diversity (high frequency) of the problem/carProblem Word-CO occurrence and high diversity (low frequency) of the solving/repair Word-CO occurrence. However, ME achieves 92.3 % %correctness for the problem-concept-EDU-boundary determination for the car-problem category as the highest %correctness in boundary determination. In addition to the car-problem category having a Problem Word-CO occurrence with low diversity, ME gives better results for the problem-concept EDU boundary determination than SVM. Furthermore, in the disease categories, ME still gives better results for the solving-concept EDU boundary determination than SVM because ME is a probabilistic classifier based on feature frequency occurrence with some feature dependencies as Problem Word-CO features, i.e. ‘*รู้สึก*/*feel* + *คลื่นใส้*/*nauseate*’ (***feel nauseated***) and ‘*อาเจียน*/*vomit* + ‘’/*null*’ (***vomit***) are dependent, whereas SVM is based on the hyperplane separation in a multidimensional feature space. Since there is high diversity for the Problem Word-CO occurrence on the disease categories, the Problem Word-CO features are then sparse, which results in LR having higher %correctness for the problem-concept-EDU-boundary determination than ME and SVM.

Table 2 shows the evaluation of the Problem-Solving relation extraction in terms of the precision and the recall based on the answer sets provided by three experts with max win voting. Table 2 also presents the medical-healthcare corpus has the higher precision in extracting the problem-solving relation by Naïve Bayes with the involvement of clustering objects and clustering features than without the clustering involvement because the corpus contains several objects of the Problem Word-CO vectors and several features of the Solving Word-CO vectors that clustering features is required to reduce features. Whereas the car-repair corpus has the lower precision in extracting the problem-solving relation by Naïve Bayes with the clustering involvement than without the clustering involvement because the car-repair corpus has an uncollected Problem Word-CO occurrence or an ellipsis Problem Word-CO occurrence which effects to clustering Problem Word-CO vectors, for example:

EDU1: “*เมื่อวาน*/*yesterdayกรุงเทพ*/*Bangkokมี*/has *ฝนตก/rainหนัก*/*heavy และน้ำท่วม*/*flood*”
  (***Yesterday***, ***Bangkok had heavy rainand flooding***.)
EDU2: “*รถของฉัน*/*carmyกระตุก*/*jerk*” (***Mycar jerked***.)
EDU3: “*ขณะ*/*while* [*ฉัน*/*I*] *ขับกลับ*/*drive บ้าน*/*home*” (***whiledriving home***.)

where: the uncollected Problem Word-CO occurrence is ‘*มีน้ำท่วม*/*have*-*flood*’ ‘’ whilst the car problems of ‘*jerk with flood*’ and ‘*jerk without flood*’ are different.

**Table 2:** The accuracy of problem-solving relation extraction

Testing corpora (500 EDUs per corpus)	Problem-solving relation extraction			
	By Naïve Bayes with clustering		By Naïve Bayes without clustering	
	Precision	Recall	Precision	Recall
Medical-healthcare corpus 197 problem features, 118 solving features	0.875	0.754	0.840	0.720
Car-repair corpus 37 problem features, 68 solving features	0.822	0.742	0.852	0.790

The reason for the low recall in determining the Problem-Solving relation is the variation of the posted problems and solving steps between people with the same topic name, for example the ‘Food Poisoning’ topic name in the medical-healthcare domain; the variation of the posted food-poisoning symptoms is shown in the following sets {‘*have a headache*’, ‘*have a colic*’, ‘*vomit*’, ‘*be dizzy*’}, {‘*have diarrhea*’, ‘*have fever*’, ‘*be nauseated*’, ‘*vomit*’}, {‘*have diarrhea*’, ‘*vomit*’}, {‘*have diarrhea*’, ‘*have a colic*’}, etc., which results in varying their actual treatments. Both the symptom variation and the actual treatment variation affects both object clusters and feature clusters in the relation learning step.

## Conclusion

In this paper, we presented the extraction of a group-pair relation between two event-explanation groups expressed by several EDUs with boundary consideration from downloaded documents. The group-pair relation that we addressed in our research is the Problem-Solving relation, i.e. a DiseaseSymptom-Treatment relation and a CarProblem-Repair relation, where disease symptoms and car problems are the problem-event explanation group, and the treatment steps and repair steps are the solving-event explanation group. With regard to the limited literation of determining the semantic relation, particularly a group-pair relation, from texts for preliminary problem diagnosis, our research extracted the group-pair relations as an explanation based relation from web-board documents for preliminary problem solving. Our proposed method of extracting the group-pair/Problem-Solving relation from texts is based on two EDU vectors, a problem-concept EDU vector and a solving-concept EDU vector, where each EDU is represented by a Word-CO feature. Each Word-CO feature consists of a verb as the first word and the second word is a co-occurring word right after the first word with either a problem-event concept or a solving-event concept. To evaluate the proposed method, the accuracy of the Problem-Solving relation extraction depends on the corpus domain and also the corpus behavior, i.e. the number of different Word-CO features, the number of Word-CO features etc. In contrast to previous works where the relations occur within one sentence or one vector of sentences, our proposed approach (based on two vectors of sentences/EDUs) enables a group-pair/Problem-Solving relation extraction with high accuracy. In the future, the ellipsis feature, the temporal feature and the condition feature should be considered to increase the accuracy of the Problem-Solving relation extraction by reducing the problem variety and the solving variety in terms of conditional groups. Moreover, the proposed method can also be applied in other languages, and the extracted DiseaseSymptom-Treatment relation including PSM representation (Fig. 6) can provide knowledge for non-professional persons to understand how to solve their problems at an earlier stage.
